# The Muriate of Cocaine—A Clinical Lecture

**Published:** 1884-12

**Authors:** E. L. Holmes

**Affiliations:** Illinois Eye and Ear Infirmary


### ARTICLE II.

THE MURIATE OF COCAINE—*A Clinical Lecture, by* DR. E. L. HOLMES, *at the Illinois Eye and Ear Infirmary.*

*Gentlemen:* — I wish to call your attention to the subject of local anæsthesia, produced by one of the active principles of South American tea. Although some of the physiological effects of this plant have been known to Europeans many years it is only quite recently that it was found to possess such remarkable properties — that the discovery must be regarded as one of the greatest in the medical history of the century.

It has been in use by laryngologists more or less for about a year. Its utility, however, seems to have made little impression upon the minds of these specialists, and much less on the minds of other practitioners.

About three months ago the discovery was made that a few drops of a two to four per cent. solution of the muriate of cocaine placed upon the conjunctiva and cornea produced in about ten minutes almost absolute anæsthesia of these tissues. Many of the most important operations have been performed upon the eye thus anæsthetized, with scarcely the slightest pain.

Important facts like these could not fail to attract the attention of the profession in every country of the world.

It is not necessary for me to dwell at this time on the medical history of the plant, nor the manner in which it has gained its high position in the list of very valuable remedies. You will find the general subject discussed in late works on Materia Medica, and the special application described in nearly all medical journals of the day.

Thus far, nearly all the reports are confined to the use of coca in ophthalmic surgery. It will probably have considerable value in facilitating operations without ether or chloroform on the mucous membrane of nearly every portion of the body—and possibly of some value in otology, in general surgery and in dentistry.

I have myself performed three operations for secondary cataract, in each of which the patient was perfectly freed from pain. A very timid, nervous lady, from whose eye a large loose piece of thick capsule was drawn by means of a camelar forceps, submitted to the operation as quietly as she could have done under the influence of ether. She did not feel the grasp of the fixation forceps, nor did she experience pain in any step of the operation. The other patients were equally free from discomfort.

I have preferred to use the few grains, which I could obtain in attempting to relieve the pain, irritation, photophobia, lachrymation and spasm of the lids—often found in phlyctenular keratitis, superficial ulcerating of the cornea.

You have before you a young man with painful ulcerative keratitis and severe spasm of the orbicularis. Five drops of a four per cent. solution was instilled between the lids at intervals of two minutes. The severe symptoms were relieved at once, and under the influence of a couple of drops morning and evening have not returned at the end of three days. The patient can open his eye quite freely, even in strong light.

You perceive also another patient, whose left eye has been closed by spasm of the lids and ulcer of the cornea. The lachrymation has excoriated the integument around the lids. Relief was given by the treatment above described. The usual remedies are now rapidly reducing the conjunctival and corneal inflammation. I have applied the coca in four private cases, with most happy results. On the other hand, I present a patient whose very painful symptoms with lachrymation have not been relieved. Possibly if the patient was placed under the influence of ether, so as to arrest the lachrymation, the solution of cocaine would be absorbed and produce the desired effect. It could then perhaps be continued with benefit.

I have recently wonderfully relieved the pain and irritation which sometimes supervene some days after extraction of cataract. Now four days have passed with no recurrence of the pain.

I am not so enthusiastic as to believe painful affections of the eye will be unknown. But one may indulge in enthusiasm over a discovery which I believe will render a large number of minor operations on the eye painless and some of the capital operations nearly so, and which will aid in relieving much distress in many diseases.

The cost of the remedy, which is now very great, will probably soon be much reduced, since there seems to be an almost exhaustless supply of the plant from which it is derived. If any observers have been disappointed, it is, I think because they have used a too weak solution. A drop of a solution composed of four grains to the drachm, instilled between the lids five times in the course of ten minutes, will ordinarily accomplish the desired result.

